# Multi-Objective Drug Design Based on Graph-Fragment Molecular Representation and Deep Evolutionary Learning

**DOI:** 10.3389/fphar.2022.920747

**Published:** 2022-07-04

**Authors:** Muhetaer Mukaidaisi, Andrew Vu, Karl Grantham, Alain Tchagang, Yifeng Li

**Affiliations:** ^1^ Biomedical Data Science Laboratory, Department of Computer Science, Brock University, St. Catharines, ON, Canada; ^2^ Scientific Data Mining Team, Digital Technologies Research Centre, National Research Council Canada, Ottawa, ON, Canada

**Keywords:** drug design, multi-objective optimization, deep evolutionary learning, graph fragmentation, variational autoencoder, protein-ligand binding affinity

## Abstract

Drug discovery is a challenging process with a huge molecular space to be explored and numerous pharmacological properties to be appropriately considered. Among various drug design protocols, fragment-based drug design is an effective way of constraining the search space and better utilizing biologically active compounds. Motivated by fragment-based drug search for a given protein target and the emergence of artificial intelligence (AI) approaches in this field, this work advances the field of *in silico* drug design by (1) integrating a graph fragmentation-based deep generative model with a deep evolutionary learning process for large-scale multi-objective molecular optimization, and (2) applying protein-ligand binding affinity scores together with other desired physicochemical properties as objectives. Our experiments show that the proposed method can generate novel molecules with improved property values and binding affinities.

## 1 Introduction

Drugs are essentially molecules with a pharmacological profile that compromises numerous relevant objectives such as potency, selectivity, pharmacokinetics, and toxicity. Drug discovery is the process of finding new therapeutically useful compounds or repurposing existing ones, with desirable pharmacological properties. After identification of a drug target (often a protein), traditional approaches to drug discovery include preparing a set of molecules with specific properties, studying the relationship between their structures and properties, and improving the compound structure. This trial-and-error-based approach is often costly and ineffective; the development of novel drugs requires billions of dollars in investment and up to decades in the development cycle (Oduguwa et al., 2006). In contrast to the traditional approaches that require iterative and collective involvement of domain experts and focus on deriving properties from structures, modern AI-driven drug discovery methods aim at efficiently searching through a vast space of molecules for promising candidates, this can be viewed as either a molecular generation problem or a molecular optimization problem.

A molecular generation problem is a generative machine learning task. It involves a generative model that can capture the regularities or patterns of the given set of molecules using a probabilistic distribution, and then generates new plausible molecules following a sampling mechanism. However, difficulties in producing high-quality novel candidates by prior generative methods arise because of the discrete nature of chemical space and the large number of molecules therein. The recent application of distributed representation methods (e.g., word or graph embedding) and deep generative models (DGMs) in drug design (Duvenaud et al., 2015; De Cao and Kipf, 2018; Romez-Bombarelli et al., 2018) enables the modelling of molecular data via a parameterized distribution *p*_***θ***_(***x***, ***z***) where ***x*** corresponds to a molecule, ***z*** is a latent vector, and ***θ*** is the set of neural network parameters. While generative models generate molecules in consistence with the training data distribution, molecular optimization on the other hand is the process of designing new molecules with the desired properties, rather than naively enumerating the entire molecular space. Molecular optimization problems can be grouped into conditional and unconditional optimization tasks. A conditional optimization task relies on the principle of local optimization given a molecule as a starting point and aims at finding structurally similar molecules with better properties, whereas an unconditional optimization task employs global optimization/search techniques. Since drug candidate needs to quantitatively fulfill multiple desiderata, molecular optimization problems are essentially multi-objective optimization problems.

While a drug-like molecule needs to fulfill physicochemical and structural feature requirements, such as Lipinski’s rule of five (Lipinski et al., 2001) as a rule of thumb for druggability, it is important that the molecule specifically binds to the expected binding site of a protein target. Using molecular mechanism simulation, protein-ligand docking is the standard mean for virtual screening. Among many other efficient open-source docking tools, Rosetta (Leman et al., 2020) and AutoDock Suite (Eberhardt et al., 2021) are widely adopted in the research community. Recently, machine learning approaches have been exploited to predict drug-target interaction (DTI) or drug-target binding affinity (DTBA) through learning on heterogeneous biological data of known interactions to understand the mechanism of drug actions. For example, AutoDTI++ (Sajadi et al., 2021) uses a denoising autoencoder that reconstructs the drug-target interaction matrix by adopting denoising empirical loss, which emphasizes interaction prediction while discarding the loss of missing values. The model input is composed by multiplying the drug-target interaction matrix by the fingerprint-drug matrix for additional information on drug fingerprints. (Abbasi et al., 2020) introduces the DeepCDA model containing a training encoder and a test encoder for cross-domain binding affinity prediction of novel drug-protein pairs. The adversarial discriminative domain adaptation (ADDA) technique is utilized in the test feature encoder to map the marginal distribution from both training and test domains into one same feature space. They also proposed a combination of convolutional neural network (CNN) and long-short-term memory (LSTM) neural network with the aid of a two-sided attention mechanism for encoding the interactions between the compound substructures and protein subsequences.

Substantially, computational methods for evaluating chemical structures must rely upon a suitable molecular representation, as the form in which a molecular structure is seen by the algorithm. One of two methods is typically applied in molecular representation: SMILES-based method and graph-based method which are discussed below.

The SMILES (Simplified Molecular Linear Input Specification) (Weininger, 1988) strings obtained by the graph-to-text mapping algorithm have been widely used for the representation of molecules. Since SMILES relies on sequence-based embedding representations, natural language processing (NLP) algorithms can naturally be ported to the field of molecule modelling. SMILES-based methods accommodate specification in the form of a line notation for describing the structure of chemical species as short ASCII strings, and often involves a variational autoencoder (VAE) (Kingma and Welling, 2014) framework as a character-based language model of SMILES strings to permit efficient molecular generation and optimization through the open-ended latent representation space (as chemical spaces) (Dai et al., 2018; Romez-Bombarelli et al., 2018). FragVAE (Grantham et al., 2022) modifies the SMILES fragment-based drug design approach from (Podda et al., 2020) that exploits the Breaking of Retro-synthetically Interesting Chemical Substructures algorithm (BRICS) (Degen et al., 2008) to collect sequences of fragments for molecules. BRICS integrates more elaborate medicinal chemistry rules and decomposes a molecule via breaking strategic bonds that can later be used for chemical motif recombination. In analogy with NLP tasks, these sequences of fragments are considered “sentences”, with each fragment as a “word”, and are embedded into continuous latent space based on a vocabulary of unique “words” (Mikolov et al., 2010, 2013). In FragVAE, Gated Recurrent Unit (GRU) (Chung et al., 2014; Li et al., 2016) based encoder is adopted to map embeddings into a stochastic latent representation space. A latent vector (either generated using the encoder or sampled from the prior distribution) is used as the initial hidden state of a GRU-based autoregressive decoder which produces the probabilities of the next possible fragments in the sequence. Using a greedy strategy for selection, the fragment sequence with highest probability is then reassembled into a molecule.

Similar to SMILES string generation, molecular graph generation usually sequentially adds nodes (atoms) and edges (bonds) to a graph. As another family of molecular generation models, graph-based methods (De Cao and Kipf, 2018; Simonovsky and Komodakis, 2018; Samanta et al., 2020) represent a molecular structure as a two/three-dimensional graph, and can operate explicitly on the molecular topology in a generator. Using VAE as a base, researchers have proposed a variety of methods to generate molecules that map directly from latent vectors. However, when VAE performs reconstruction, it is computationally expensive to solve the problem of graph isomorphism. Thus, the effectiveness and accuracy of graph reconstruction are extremely low without imposing constraints. Currently, one of the most successful approaches to transforming molecular graphs into meaningful latent vectors and avoiding sequential generation is the junction tree variational autoencoder (JTVAE) (Jin et al., 2018), which we view as graph fragment-based based method (Pogány et al., 2019). JTVAE decomposes training molecules into a set of molecular substructures including rings, functional groups, and atoms. Compared to the conventional node-by-node generation of a graph, JTVAE assembles these building blocks in a two-stage generation process: (1) representing the effective brackets and their arrangement as a scaffolding tree, and (2) integrating the whole tree into a graph by adding edges between intersecting components. The authors of JTVAE also improved JTVAE with graph-to-graph transformation (Dai et al., 2016; Gilmer et al., 2017) and autoregressive methods (Kusner et al., 2017; Mueller et al., 2017) to enable molecular property optimization. The latest development in graph-based molecular modelling include 3D-graph and geometric deep learning methods to take advantage the geometric properties of molecular structures (Atz et al., 2021; Luo et al., 2021).

Developed for multi-objective molecular optimization, the deep evolutionary learning (DEL) framework (Grantham et al., 2022) proposes innovation in extending metaheuristic multi-objective global optimization methods (e.g., multi-objective evolutionary computation (Deb et al., 2002; Eiben and Smith, 2015) to their corresponding deep versions through the latent representation space of DGMs. DEL achieves the co-evolution of both molecular data and molecular generative models across multiple generations guided by multiple properties concerned with drug design. The DGM used in the original DEL is the SMILES fragment-based model, FragVAE. Even though the integration of FragVAE in DEL achieves interesting results in drug design, the BRICS strategy in FragVAE usually splits a molecule into only 2-4 fragments, which may lead to issues in language models due to this level of coarse granularity. While we continue looking for SMILES fragmentation methods in finer granularity, we are interested in investigating graph fragmentation methods for molecular representation in DGMs and DEL.

In this paper, encouraged by past success of target-specific fragment-based drug design methods and the recent development of AI techniques for drug design, we present an improvement of DEL using the graph fragment-based method, JTVAE, as the deep generative model for multi-objective molecular optimization. Specifically, our work has two major contributions. First, we introduce the embedding and generation of molecular graph fragments to DEL through incorporating the graph-fragmentation deep generative model, JTVAE. We compared this graph fragment-based DEL with the SMILES fragment-based DEL developed in (Grantham et al., 2022) in our framework, and found that the JTVAE-based DEL is able to perform better than the FragVAE-based DEL in terms of molecular quality.

As our second contribution, we apply the protein-ligand binding affinity score as one of the molecular optimization objectives in DEL, while the original DEL in (Grantham et al., 2022) entails drug-likeness and synthesizability. The majority of drug compounds take effect by specifically binding to active sites of the specified protein targets responsible for diseases (such as COVID-19, AIDS, cancer, autism, Alzheimer’s, etc.), making drug design a challenging task for medicinal chemists. When the target protein structure becomes accessible, molecular docking is more frequently involved *in silico* drug design to assess potential protein-ligand binding interactions (Walters et al., 1998; Pagadala et al., 2016), where the ligand is usually a molecule that forms a complex with its receptor to modify pathways associated with diseases. In this work, protein-ligand binding scores of molecules indicate their binding affinity toward a preeminent target protein surface area, and are calculated using molecular mechanism simulation. Aiming at identifying active drug candidates, the binding score is viewed as a desired property along with other choices of objectives to be optimized in DEL.

## 2 Methods

The idea of our approach is to integrate the graph fragment-based deep generative model, JTVAE, into the DEL framework, such that JTVAE provides a latent representation space for the multi-objective evolutionary algorithm (MOEA) to explore, and meanwhile elite samples from each generation of MOEA are used to improve the continual learning of JTVAE. The protein-ligand binding affinity score (denoted by BAS) (Lipinski et al., 2001; Engel and Gasteiger, 2018), synthetic accessibility score (SAS), and water-octanol partition coefficient (logP) of molecules are used as three objectives in the optimization. In the following subsections, the general framework of DEL and two DGMs, FragVAE (used as a benchmark) and JTVAE, are described in detail.

### 2.1 Deep Evolutionary Learning Framework

**FIGURE 1:**
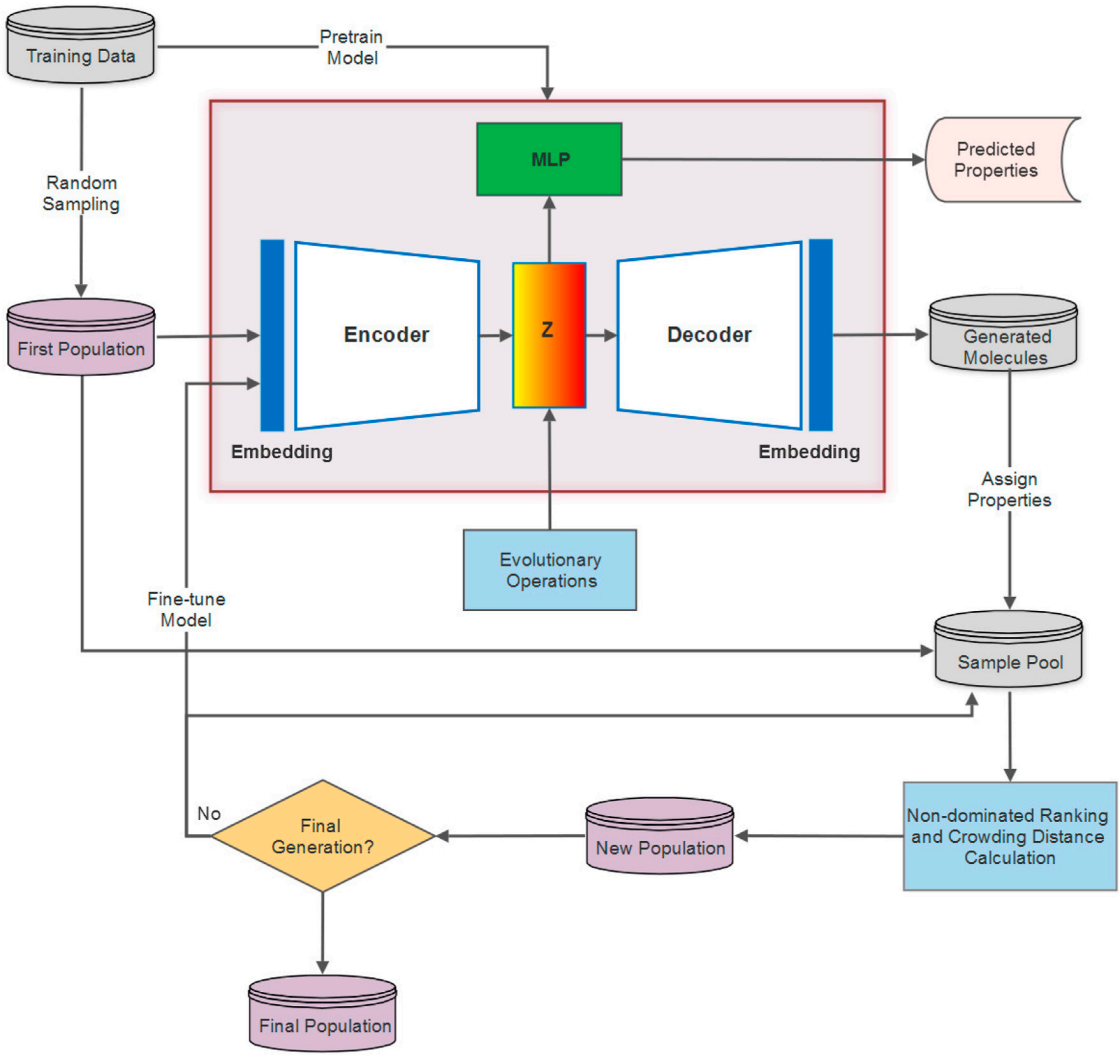
Diagram showing the workflow of the DEL framework and its interaction with VAE.

Proposed in (Grantham et al., 2022), the DEL framework incorporates multi-objective evolutionary computation with the deep generative models for molecular optimization by establishing a data-model co-evolution paradigm. In contrast to traditional evolutionary algorithms that encode genotypes in the original problem space, DEL instead employs evolutionary operations in the latent representation space of molecules to feedback the evolved data with desired properties, leading to the fine-tuning of the deep generative model as well. The DEL framework comprises the following components. (1) For the first evolutionary generation, the DGM (usually a VAE) is parameterized and pretrained on the training data, and the first population is sampled from the original training data; whereas the population samples of the successive generations are obtained from the DGM. (2) The samples are transformed into latent vectors with the help of an encoder. Meanwhile, the samples are processed to assign molecular properties and are subject to a multi-objective sorting method (e.g., non-dominated ranking) and crowding distance computing. (3) Evolutionary operations are applied to the latent representations of population samples considering their Pareto ranks and crowding distances. This is a randomized and stochastic technique that simulates the evolutionary process of selecting high-fit candidates and exerts evolutionary operators entailing “crossover” and “mutation” to evolve better molecules (Nicolaou et al., 2009). (4) The evolved latent representations are decoded by the DGM to generate new molecules, which are then evaluated on the basis of validity, novelty, and uniqueness using RDKit (Landrum, 2006). Invalid and duplicate individuals are eliminated to form new samples for new population construction. (5) The new population of each generation is constructed of the high-quality valid samples from the previous population and newly generated data in Step (4), and can also be used to fine-tune the DGM. (6) Steps (2–5) are repeated for multiple generations as needed to compose the final population. The specific interaction between the DEL framework and DGM is illustrated in Figure 1.

High-quality samples for fine-tuning the VAE model are selected based on the non-dominated ranking result, which underlines the molecules with the most valuable properties in terms of SAS, logP, and BAS. Of course, our approach also operates on other properties together with the protein-ligand binding affinity score, that is, generated molecules with smaller SAS, logP, and BAS are prioritized in ranks and exploited in the evolutionary computation process. SAS and logP are calculated using RDKit; BAS is produced by QuickVina (Alhossary et al., 2015).

### 2.2 Deep Generative Model

In this work, two different forms of VAE (FragVAE and JTVAE) are employed as the DGM in the DEL framework. In terms of structure, VAE can be viewed as a variant of the autoencoder (AE) architecture (Hinton and Zemel, 1993) which is a deterministic neural network involving an encoder and a decoder that are learned unsupervised. The learning objective of AE is to reconstruct the input ***x*** (to the encoder) using the decoder output $\tilde{x}$ . The hidden layer ***z*** between the encoder and decoder generates a code to represent the input. Thus, the network can be seen as a harmony of two parts: an encoder represented by the function ***z*** = *f*(***x***) and a decoder $\tilde{x}=g\left(z\right)$ that generates reconstructions. An AE is not particularly useful if it simply learns to set *g* (*f*(***x***)) = ***x*** everywhere because, conversely, it should not produce output identical to the input. This usually imposes some constraints or regularizations on the AE so that it generates approximately similar data. These constraints force the model to consider which parts of the input data need to be preferentially replicated. Thus, it tends to learn beneficial features of the data.

VAE uses deep neural networks to parameterize the generative component *p* (***x*** | ***z***) and inference component *q* (***z*** | ***x***) in a generative model, thus forming a family of deep generative models. Even though the latent vector (i.e., the bottleneck of the architecture) is stochastic, the application of the reparameterization trick enables the use of gradient descent for model learning. In VAE, the encoder (inference) network can be defined as *q*_***ϕ***_(***z*** | ***x***) parameterized by ***ϕ*** and decoder (generative) network *p*_***θ***_(***x*** | ***z***) with learnable parameters ***θ***. In the training process, the encoder maps input ***x*** to the stochastic latent vector ***z*** which is then passed to the decoder to generate the reconstruction $\tilde{x}$ . Based on this, the following negated variational loss is formulated for minimization:

$$L\left(\phi ,\theta \right)=-E_{z∼q_{\phi }\left(z|x\right)}\left[\log p_{\theta }\left(x|z\right)\right]+KL\left(q_{\phi }\left(z|x\right)\|p\left(z\right)\right).$$ (1)

The first term above is the reconstruction loss, which is a process through $x∼z∼\tilde{x}$ , and can also be described as the expected negated log-likelihood. The second term is a regularization term using Kullback-Leibler (KL) divergence to measure the proximity between posterior *q*_***ϕ***_(***z*** | ***x***) and prior *p*(***z***). In practice, the prior *p*(***z***) is usually assumed to be a standard multivariate Gaussian distribution *N* (**0**, ***I***). After model training, the latent variable ***z*** at the bottleneck is expected to be approximate to the standard Gaussian distribution. Thus, disparate data can be generated by sampling from this distribution and passing through the decoder network.

#### 2.2.1 FragVAE Integration

FragVAE was implemented as a DGM in DEL for drug design based on SMILES fragmentation. Fragment-based drug design (FBDD) (Shuker et al., 1996; Erlanson, 2011) is established as an alternative approach to atom-and-bond methods, and demonstrates constructive outcomes. FBDD appropriates fragmentation of molecular structures as the screening method that breaks molecules into small-weighted components. The advantages of molecular fragmentation are threefold. (1) Small organic molecules, corresponding to the fragments, are efficiently synthesizable, hence easier to manipulate chemically. (2) Since drug-like molecules may share analogous fragments, fragmentation can help identify components that are possibly responsible for biological activities. And, (3) it can drastically reduce the search space for exploration and characterization. Moreover, by incorporating FragVAE with DEL and using the protein-ligand binding affinity score as one of the objectives, it may lead to the discovery of highly potential drug candidates. Therefore, FragVAE is investigated in this paper and compared with the graph fragment-based method, JTVAE.

**FIGURE 2:**
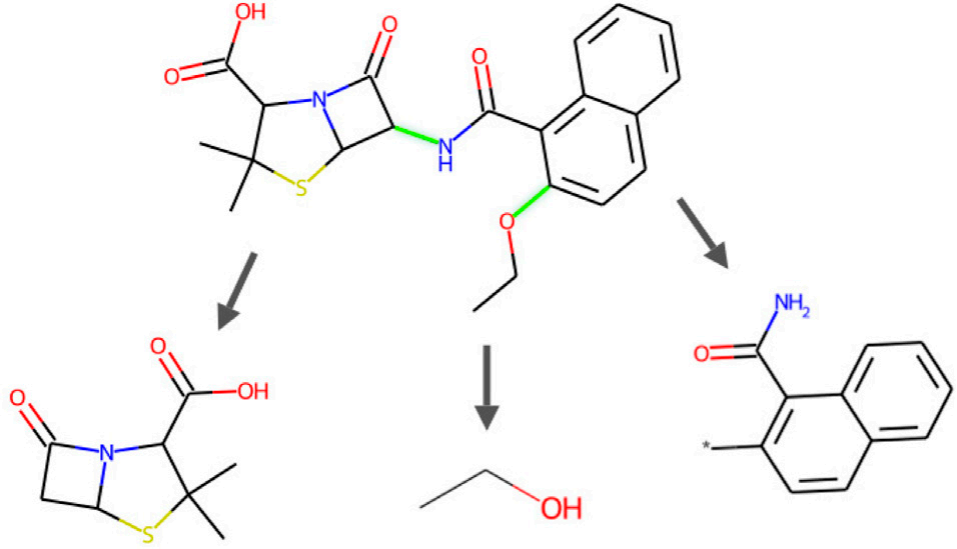
Cleavage example of an FDA-approved small-molecule durg Nafcillin (a penicillin derivative antibiotic, DrugBank Access Number DB00607), demonstrating the procedure of BRICS-based fragmentation by producing fragments on breakable bonds. In this example, the SMILES representation is CCOc1ccc2ccccc2c1C(=O)NC1C(=O)N2C1SC(C)(C)C2C(=O)O, and is broken into fragments *0CC, *NC(=O)c1c(*)ccc2ccccc12, and *C1C(=O)N2C1SC(C)(C)C2C(=O)O.

In terms of the fragmentation procedure, given a SMILES string, the atoms are scanned from left to right, such that a fragment is extracted whenever encountering a breakable bond following the BRICS rules. This process is repeated until the remaining part cannot be split further. As an example, the fragmentation of a SMILES string is shown in Figure 2. To reconstruct a molecule, fragments can be reassembled starting from the leaves to the root, right to left.

There are two features that impact FragVAE’s integration with DEL. First, in contrast with the variational loss of the vanilla VAE in Eq. 1, a trade-off weight *β* (Higgins et al., 2017; Yan et al., 2020) is added between the KL-divergence and the reconstruction error to balance their discrepancy in minimization. Second, a multi-layer perceptron neural network (MLP) is added to the VAE structure as a property predictor (Yang et al., 2019). It intends to regularize the latent representation space by predicting the property values of encoded samples. The variational loss of the VAE model is thus altered by adding a third term for property regression error (see Eq. 2).

$$L\left(\phi ,\theta \right)=-E_{z∼q_{\phi }\left(z|x\right)}\left[\log p_{\theta }\left(x|z\right)\right]+\beta KL\left(q_{\phi }\left(z|x\right)\|p\left(z\right)\right)+\alpha E_{z∼q_{\phi }\left(z|x\right)}\left[\mathrm{SE}\left(f_{\psi }\left(z\right),y\right)\right],$$ (2)

where *f*_***ψ***_(***z***) denotes the predicted property value, ***ψ*** is the parameter set of the MLP subnetwork, ***y*** is the actual property value, and SE stands for squared error.

#### 2.2.2 JTVAE Integration

Despite the proliferation of SMILES-based models in recent years for molecular modelling, it still faces two critical limitations. (1) The SMILES syntax is not robust to small changes or mistakes, which can result in the generation of invalid or drastically divergent structures. (2) The unstructured nature of SMILES implies that two structurally similar molecules can have completely different SMILES representations. These shortcomings result in a lack of diversity and effectiveness in the resulting molecules. The graph-based deep generative models are therefore brought to the spotlight as an alternative strategy, permitting the search for the topology of molecules and their fragments. It involves a more intuitive way to represent a molecule as a graph based on its Lewis structure. Given a graph representation G=(V,E) , a node $v_{i}\in V$ represents an atom and edge $\left(v_{i},v_{j}\right)\in E$ for a chemical bond. The nodes and edges are respectively labelled and characterized according to atom types and chemical bond types. Numerous molecular graph models, such as graph neural network (GNN) (Scarselli et al., 2009), graph convolutional network (GCN) (Duvenaud et al., 2015; Kipf and Welling, 2017), message passing neural network (MPNN) (Gilmer et al., 2017), and many other methods have been explored and shown outstanding performance in molecular property prediction tasks, and consequently laid the foundation for graph-based molecular generation.

**FIGURE 3:**
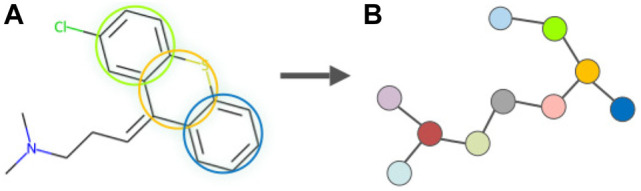
Illustration of the graph fragmentation in JTVAE following the subgraph-by-subgraph strategy, representing the process of tree decomposition on an FDA-approved small molecule drug Chlorprothixene (a thioxanthene antipsychotic, DrugBank Access Number DB01239). Clusters in the molecule **(A)** are identified as substructures and denoted as nodes in the junction tree **(B)**.

As one of the most representative VAE-based graph generative models, JTVAE (Jin et al., 2018) employs a subgraph-by-subgraph manufacturing mode instead of an atom-by-atom mode. This is to avoid revising chemically invalid intermediaries while constructing a molecular graph sequentially atom-by-atom, allowing the model to consistently generate valid molecules since validity is checked at each step following a non-sequential method. In JTVAE, a cluster vocabulary is first constructed containing simple rings, bonds, and atoms. The molecular graph *G* is first scanned to label out the substructures that appear in the vocabulary and the edges not belonging to any cycles. Two simple rings are merged as bridged compounds if they have three or more overlapping atoms. This step eradicates the cycles in molecular structures by considering them as clusters. A graph of clusters is constructed by adding edges between all intersecting clusters and composited into a junction tree by mapping its maximum spanning tree. Tree nodes in the junction tree associate the vertices in the cluster graph, and the connectivity between nodes corresponds to the chemical bonds between clusters. Figure 3 illustrates the graph fragmentation using an FDA-approved drug.

As in FragVAE for DEL, the same modification pattern is applied to JTVAE when integrated with the DEL framework. An MLP property predictor is added to the generative model and thus an SE loss term is appended to the model’s total loss. We use the two-part latent representation ***z*** = [***z***_*T*_, ***z***_*G*_] introduced in (Jin et al., 2018) with ***z***_*T*_ as the map of the junction tree structure of a molecule on the cluster level and ***z***_*G*_ as the encoding of the association within each cluster. Considering the tree decomposition of a molecule, the architecture of the modified JTVAE consists of the following five components: (a) a graph encoder *q* (***z***_*G*_|*G*) to encode molecular graph *G* to its latent representation ***z***_*G*_, (b) a tree encoder *q* (***z***_*T*_|*T*) to encode the junction tree *T* decomposed from molecular graph to acquire the latent vector ***z***_*T*_, (c) a property predictor for the latent space regularization, (d) a tree decoder *p* (*T*| ***z***_*T*_) to decode the junction tree from ***z***_*T*_, and (e) a graph decoder *p* (*G*| ***z***_*G*_) to eventually manifest the molecular graph. The tree message passing neural network (TMPNN) (Gilmer et al., 2017) and the graph message passing neural network (GMPNN) (Dai et al., 2016) with GRU units are used in the transformation of representations. The reconstruction error in JTVAE becomes

$$R=L_{c}\left(T\right)+L_{g}\left(G\right)+L_{s},$$ (3)

where *L*_*c*_(*T*), given junction tree *T*, is the entropy loss of the tree decoder, that is the summed error of topological prediction (binary prediction of the existence probability of child node) and label prediction (label of generated child node); *L*_*g*_(*G*) and *L*_*s*_ are, respectively, the negative expectations of log-likelihood of subgraph prediction based on tree nodes, and stereoisomer prediction by comparing the cosine similarity of its graph representation. Therefore, the overall loss function of the modified JTVAE is defined as:

$$\begin{array}{ll} L\left(\phi ,\theta \right) & =-E_{z∼q_{\phi }\left(z|x\right)}\left[R+\beta KL\left(q_{\phi }\left(z|x\right)\|p\left(z\right)\right)\right]+\alpha E_{z∼q_{\phi }\left(z|x\right)}\left[\mathrm{SE}\left(f_{\psi }\left(z,y\right)\right)\right],\\ z & =\left[z_{T},z_{G}\right],\\ q_{\phi }\left(z|x\right) & =\left[q_{\phi }\left(z_{T}|T\right),q_{\phi }\left(z_{G}|G\right)\right]. \end{array}$$ (4)

### 2.3 Protein-Ligand Binding Affinity Score Calculation

Implementing a reliable and suitable docking score calculation and exporting module is essential to integrate protein-ligand BAS within DEL for molecular optimization. In this respect, we adopted the efficient docking tool Quick Vina (QVina) (Alhossary et al., 2015) which is an amended version of the well-known AutoDock Vina tool (Trott and Olson, 2010; Eberhardt et al., 2021). AutoDock Vina is an open-source molecular docking engine in the AutoDock suite, and shows dominant performance in virtual screening by adopting the benchmark data Directory of Useful Decoys (DUD). While AutoDock Vina is accurate, it is proven to be time-consuming due to the exhaustiveness of the search on the 3D target surface. Hence, with an improved search algorithm that performs faster and yet still accurate BAS calculation, QVina turns out to be a better choice for our research. The docking in this work is conducted on carbonic anhydrase IX (CA9/CAIX) protein (Span et al., 2003), which has been used as a prominent marker of tumour hypoxia with the potential to serve as a diagnostic biomarker, prognostic indicator, and tumour therapeutic target. The binding site coordinate and constrained search box size in the docking score calculation module are user-specified. For our experiments, generated molecules from both methods are docked to a cubic box of size 60 centred at coordinate [9.879, -13.774, 7.012] in the binding pocket. When sufficient experimental data for CA9 become available in the future, machine learning-based approaches will be investigated for protein-ligand binding affinity prediction.

### 2.4 1-Wasserstein Distance

In the space R , there are many ways to describe the distance between two probability distributions *q*(*x*) and *p*(*x*). One of the more popular ones is KL divergence:

$$KL\left(p∥q\right)=\int_{R}p\left(x\right)\log\frac{p\left(x\right)}{q\left(x\right)}dx.$$ (5)

As defined above, KL does not measure the geometric properties of R , because the comparison between *q*(*x*) and *p*(*x*) is made at the same points. This motivates us to utilize Wasserstein distance (WD) as a distance metric for a pair of distributions. For the probability distributions *μ* and *υ* defined on R , the *p*-th Wasserstein distance is given as:

$$W_{p}\left(\mu ,\upsilon \right)≔\inf_{\gamma \in \Gamma \left(\mu ,\upsilon \right)}{\left(\int_{R\times R}d{\left(x,y\right)}^{p}d\gamma \left(x,y\right)\right)}^{\frac{1}{p}},$$ (6)

where Γ(*μ*, *υ*) is the collection of joint probability measures *γ* on R×R with marginal distributions *μ* and *υ*. A measure with marginals *μ* and *υ* is also called the coupling of *μ* and *υ*. *d* can be any distance on R , such as Euclidean distance, *l*_1_ distance, etc. In the case of *p* = 1 and *d*(*x*, *y*) = |*x* − *y*|, the one-dimensional Wasserstein distance (1-Wasserstein distance) is explicitly formulates as:

$$W_{1}\left(\mu ,\upsilon \right)=\int_{R}|F_{\mu }\left(x\right)-F_{\upsilon }\left(x\right)|dx,$$ (7)

where *F* is a cumulative distribution function.

### 2.5 Hypervolume Measure

In multi-objective evolutionary algorithms, a necessary condition for algorithm convergence is an extremely important aspect. DEL retains the elite solution set of the previous generation and adds it to the evolutionary process of the new generation. The solution set of the evolutionary population continues to converge to the real Pareto Frontier, and finally, acquires a satisfactory optimization solution. Usually, when analyzing the performance of a multi-objective optimization algorithm, we hope that the algorithm can advance in three aspects. (1) The distance between the real Pareto front surface and the one obtained by the algorithm should be as small as possible. (2) Although the obtained individual solution points are only partial solutions, they should distribute on the Pareto front as uniformly as possible. And, (3) a sufficient number of solution points should be able to cover the entire front, that is each region on the front should be represented by solution points unless this region is missing on the actual optimal Pareto front. The hypervolume (HV) index measures the volume of the dimensional region in the target space bounded by the non-dominated solution set obtained by a multi-objective optimization algorithm and a pre-specified reference point. The mathematical representation of the HV calculation is given in Eq. 8:

$$HV=\delta \left(\cup_{i=1}^{\left|S\right|}v_{i}\right),$$ (8)

where *δ* represents the Lebesgue measure, which is used to compute volume, |*S*| represents the number of non-dominated solutions, and *v*_*i*_ represents the hypercube formed by the reference point and the *i*-th solution in the solution set. HV is an effective quantitative scalar metric, which is strictly monotonic in terms of Pareto dominance. The larger the value of HV, the better the performance of the corresponding algorithm. Especially, the calculation of the HV index does not require the ideal Pareto front of the test problem, which greatly facilitates the use of HV in practical applications.

## 3 Experiments

### 3.1 Data

**FIGURE 4:**
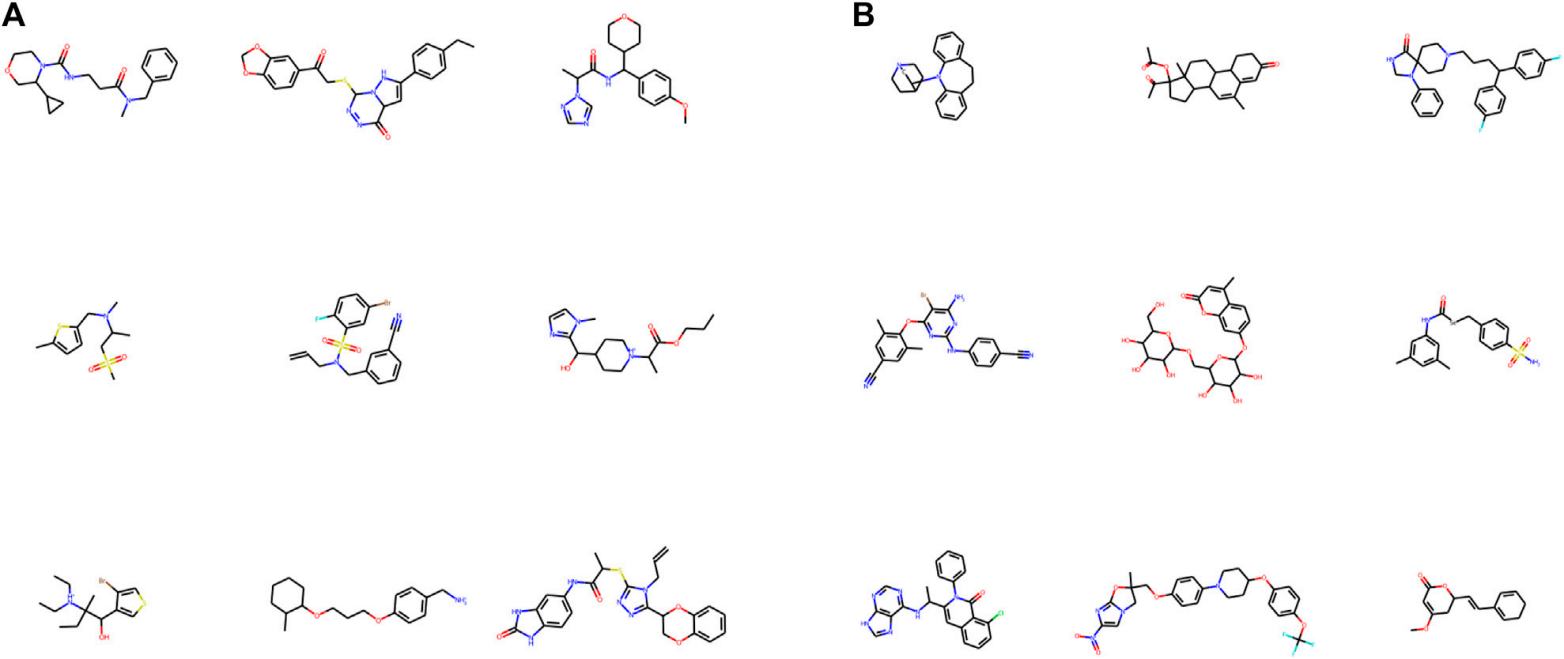
2D graph visualization of randomly chosen molecules from ZINC **(A)** and DrugBank **(B)** datasets. RDKit was used for visualization.

The experiments were conducted on the ZINC dataset (Irwin and Shoichet, 2005) and a variant of ZINC by appending authentic drug molecules from the DrugBank database (Wishart et al., 2018) (named as ZINC+DrugBank hereafter). The ZINC dataset is a popular benchmark set for generative tasks that comprise approximately 250K molecules in SMILES string format. DrugBank is a web-based database hosting detailed information on medicines including identification, pharmacology, interactions, properties, and clinical trials. We formed a subset by extracting 1932 small-molecule drugs from DrugBank and fused them into the original ZINC dataset. Molecular samples drawn from the original ZINC and DrugBank data are visualized in Figure 4.

The ZINC and DrugBank datasets were both subjected to a three-fold preprocessing step. (1) For FragVAE, molecules are cleaved into SMILES fragments following the BRICS algorithm, whereas in JTVAE, subgraph enumeration and tree decomposition are performed. (2) Calculation of the molecular properties (including objectives SAS, logP, and BAS) using RDKit and the protein-ligand binding score calculation module. And, (3) removal of duplicated molecules as well as molecules with fewer than 2 fragments.

### 3.2 Hyperparameter Settings

During the experiments, we used the same evolutionary learning hyperparameters for evaluating the DEL framework, but different hyperparameter tuning for FragVAE and JTVAE to maximize their performance. Table 1 lists our key hyperparameter settings.

**TABLE 1:** Hyperparameter settings of the DEL process and DGMs respectively.

Hyperparameter	DEL	Hyperparameter	DGM	
			FragVAE	JTVAE
Number of generations	10	Embedding size	128	128
Polulation size	20000	Number of recurrent layers	2	1
Initial number of epochs	20	Hidden state dimension	128	450
Subsequence number of epochs	10	Latent space dimension	64	64
Scheduler annealing rate	0.8	Learning rate	0.0001	0.0001
Tournament selection probability	0.95	Batch size	128	32
Mutation rate	0.01	KL divergence weight *β*	0.1	0.1

### 3.3 FragVAE Versus JTVAE in DEL

The two base DGMs in DEL and two datasets lead to a set of four experiments: (1) FragVAE in DEL framework trained on the original ZINC data, (2) FragVAE in DEL on ZINC+DrugBank data, (3) JTVAE in DEL on ZINC data, and (4) JTVAE in DEL on ZINC+DrugBank data. Whenever DEL was trained on the ZINC+DrugBank data, the actual drug molecules from DrugBank were added to the initial population composing the training data of the subsequent generation.

Firstly, the performances of FragVAE- and JTVAE-based DEL were measured using three metrics: validity (the ratio of chemically valid generated molecules in the population), novelty (the ratio of validly unique generated samples that are not originated from the training dataset) and uniqueness (the ratio of generated molecules that are not duplicated in the population). Chemical validity checking was achieved by RDKit. All these metrics were scored based on the SMILES strings. Performance of DEL based on these two DGMs are reported in Table 2, which conveys that the JTVAE generated all valid molecules, and almost all samples in the last populations generated based on FragVAE and JTVAE, respectively, are novel. Moreover, both methods could maintain a highly diverse population in DEL.

**TABLE 2:** Performance metrics of the final (10^th^) population from DEL using FragVAE and JTVAE, respectively, on two datasets.

Model	Dataset	Validity	Novelty	Uniqueness
FragVAE+DEL	ZINC	0.981	0.999	0.912
JTVAE+DEL	ZINC	1	0.985	0.939
FragVAE+DEL	ZINC+DrugBank	0.962	0.999	0.959
JTVAE+DEL	ZINC+DrugBank	1	0.988	0.939

**FIGURE 5:**
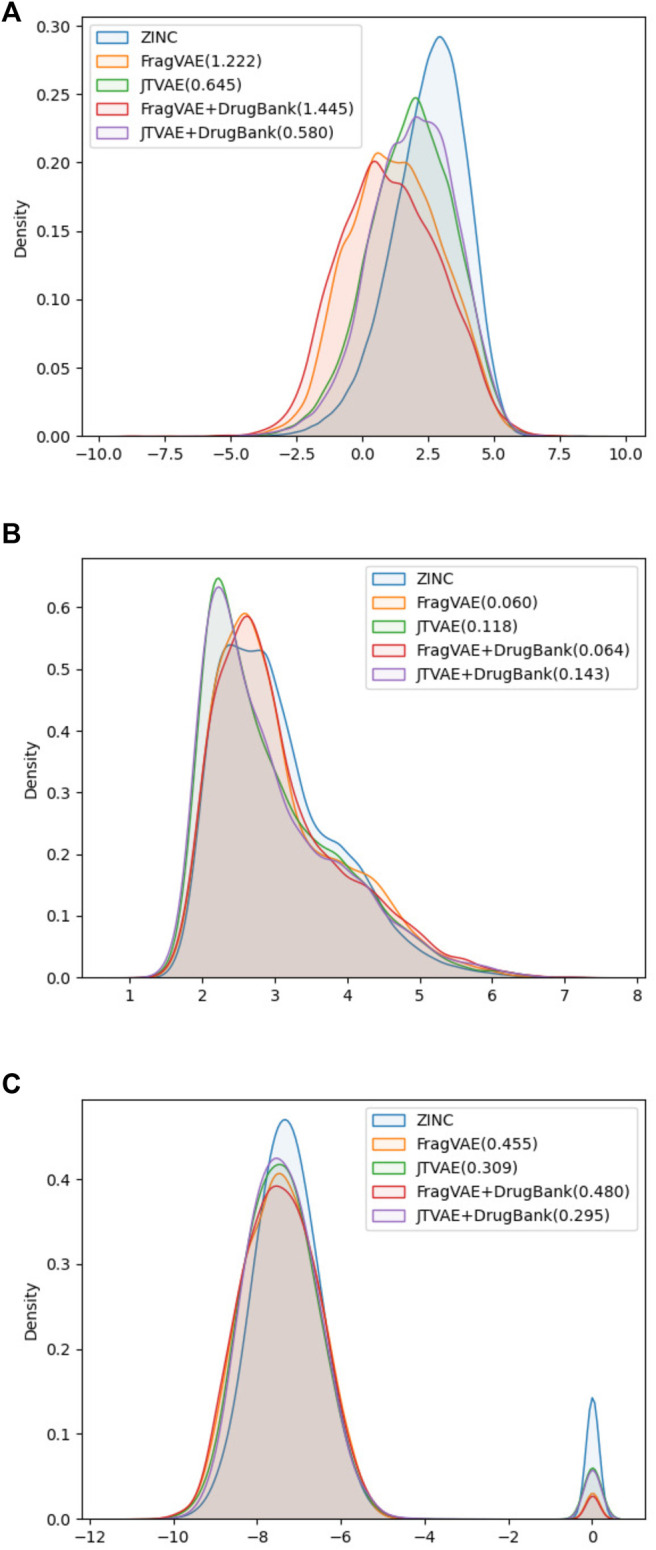
Property distributions on SAS **(A)**, logP **(B)**, and BAS **(C)** with 1-Wasserstein distances between the final population (10^th^) of DEL and the original ZINC data.

Secondly, the methods were evaluated according to the property-wise distributions of samples in their last populations (See Figure 5). Given the goal of expressing a more intuitive and quantitative comparison, the 1-Wasserstein distances (WD) from the final population of FragVAE- and JTVAE-based DEL, respectively, to the original ZINC data were calculated and indicated in the legends. It shows that both methods succeed in improving the properties through generations. While there is a slight difference between the two methods on binding affinity score, JTVAE exhibits superior performance on logP whereas FragVAE improves SAS the most.

Thirdly, while the property-wise distributions presented above can only offer a partial comparison, the Pareto-fronts obtained by DEL using different base DGMs are compared in terms of HV to reflect the overall quality of solutions. In our experiments, the number of generations was set to 10 and the trade-off hyperparameter *β* was set to 0.1. The obtained HVs in the initial generation, the middle generation, and the final generation are listed in Table 3 which demonstrates the following key features. (1) HV increases along with evolutionary generations, showing that DEL can gradually improve the quality of Pareto fronts. And, (2) the JTVAE in the DEL framework results in a higher HV value compared to FragVAE, revealing a better comprehensive performance of the graph fragmentation algorithm in DEL.

**TABLE 3:** Hypervolumes of DEL’s Pareto fronts (*β* = 0.1). The results are collected from the populations of Generations 1, 5, and 10, respectively.

Model	Dataset	Hypervolume		
		Generation 1	Generation 5	Generation 10
FragVAE	ZINC	458.87	466.91	472.88
JTVAE	ZINC	477.14	477.08	482.99
FragVAE	ZINC+DrugBank	452.01	452.52	463.76
JTVAE	ZINC+DrugBank	455.57	472.16	473.06

### 3.4 Virtual Screening

Generated samples on the first Pareto front of the final population are viewed as high-rank molecules. As we set the population size to 20,000 in our experiments, DEL usually results in 20–30 Pareto ranks. Only the first fronts are investigated in this section. We applied the following threefold criteria to identify high-quality novel samples of the first front:

• SAS ≤ 3
• − 0.4 ≤ logP ≤ 5.6
• BAS ≤ −6.6

**FIGURE 6:**
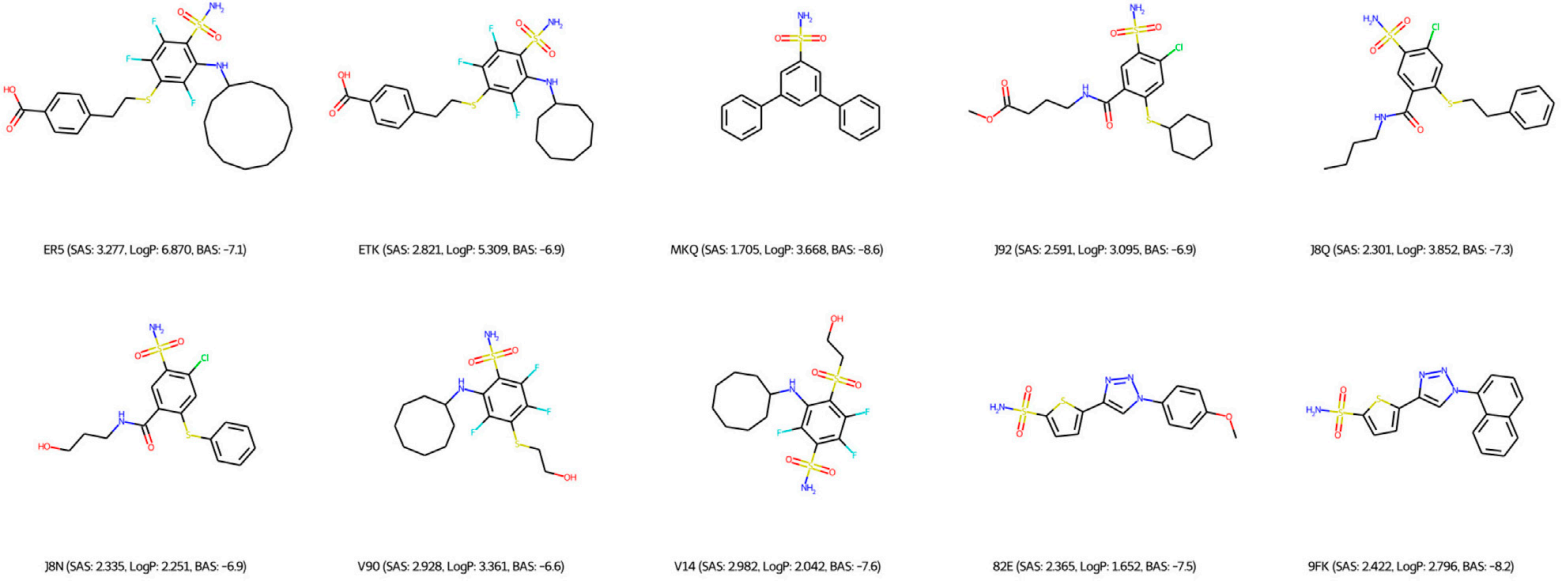
2D graph visualization of 10 unique CA9 ligands from PDB.

**FIGURE 7:**
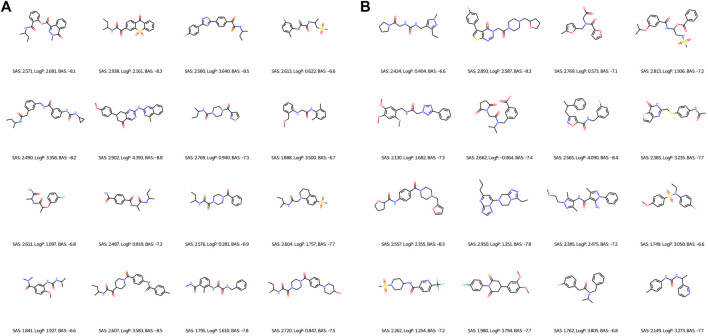
2D graph visualization of high-quality novel samples of the final (10^th^) population of DEL in combination with FragVAE **(A)** and JTVAE **(B)**, respectively, on the **ZINC** data. Due to space limit, only 16 molecules are shown for each method.

**FIGURE 8:**
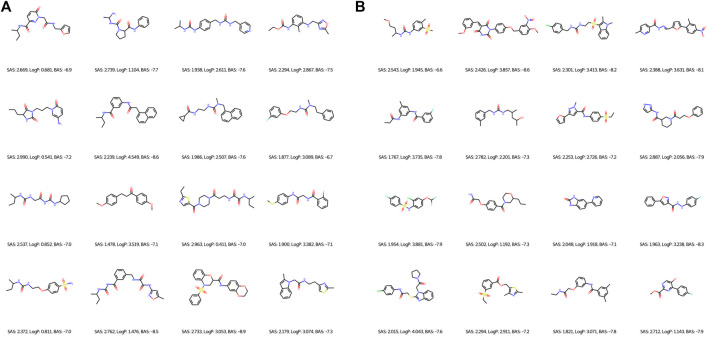
2D graph examples of high-quality novel samples of the final (10^th^) population of DEL in combination with FragVAE **(A)** and JTVAE **(B)** respectively on the ZINC+DrugBank data. Due to space limit, only 16 molecules are shown for each method.

Regarding the qualitative characterization of logP, the Ghose filter rules (Ghose et al., 1999) were considered to benefit the prediction of drug-likeness. We then identified the BAS threshold by assessing 10 unique real ligands of the target protein CA9. These ligands were collected from Protein Data Bank (PDB) (Berman et al., 2000) and processed using the scoring module as our framework to isolate the impact of docking configurations. In Figure 6, we observe a maximum BAS of −6.6 and a minimum of −8.6, thus setting the upper bound for filtering the high-quality novel molecules from the first fronts (one of our objectives is to minimize BAS). Respectively, 89 and 99 molecules were retrieved from FragVAE+DEL trained on the ZINC and ZINC+DrugBank data; 94 and 107 molecules from JTVAE+DEL were trained on the ZINC and ZINC+DrugBank data as shown in Figures 7, 8.

**FIGURE 9:**
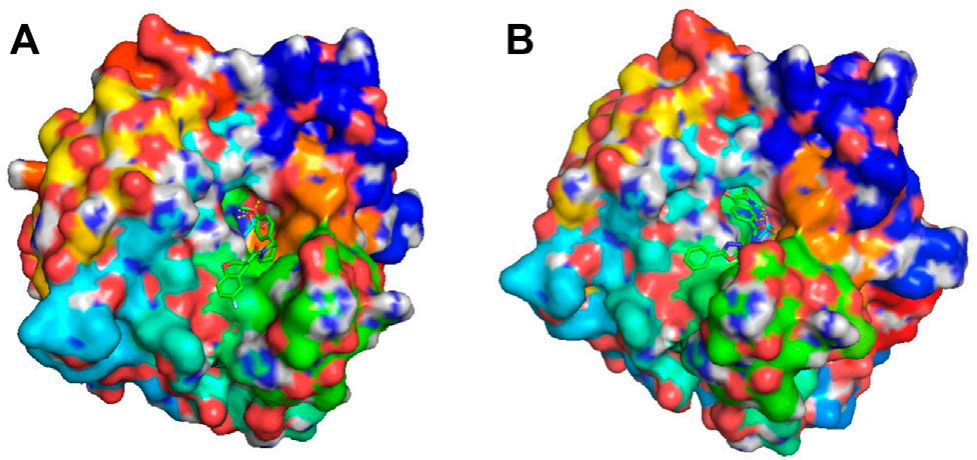
Docking visualization of two novel molecules binding on CA9 protein surface. Both molecules ranked top on BAS in the high-quality samples of their final population. **(A)** shows the molecule COc1ccc (C2CCN(C(=O)C3COc4ccc(F)cc4C3)C2)cc1 binding to the binding site of CA9 protein. It was generated by FragVAE+DEL and has a binding affinity score of −9.3 (SAS: 2.892, logP: 3.402). **(B)** shows the molecule O=C1Nc2cc(C(=O)NCCc3nnc(-c4ccccc4)o3)ccc2C1=O binding to the binding site of CA9. It was discovered by JTVAE+DEL and has a binding affinity score of −8.1 (SAS: 2.562, logP: 1.844).

Different from the traditional procedure in virtual screening which investigates the binding affinity scores of given molecules in a fixed library, our approach integrates virtual screening in the generation and optimization process through the use of binding affinity score along with other concerned objectives. The benefit of our approach is that it can find novel molecules which potentially satisfy all applied criteria. As a case study to demonstrate, we ranked the high-quality molecules descendingly by BAS and selected two novel molecules with the finest score, one was obtained using FragVAE+DEL and one from JTVAE+DEL. Figure 9 displays, thanks to PyMOL (Schrödinger, 2015), the corresponding protein-ligand complexes. As compared to the existing ligands in Figure 6, both molecules have no violation of the criteria and excel in all three objectives. For further validations, all novel molecules from the DEL results in preferred ranges of properties and binding scores can be promoted.

## 4 Conclusion

Drug discovery can be modelled as a multi-objective optimization problem over a vast search space. The advantages of target-aimed fragment-based drug design and the powerful representation and modelling capacity of deep learning methods motivated our research. We propose to apply graph fragment-based deep generative models in the deep evolutionary learning process and use the protein-ligand binding affinity score as one of the objectives. Our experiments show that our approach is able to generate novel molecules possessing better quality in terms of Pareto front hypervolume and number of novel samples satisfying the screening criteria when compared to a SMILES fragment-based deep generative model previously used in the deep evolutionary learning framework. Since both approaches use VAE and DEL, it is likely that the junction-tree based graph fragmentation contributes to the performance improvement. Furthermore, the incorporation of binding affinity score as one of the objectives enables us to identify and list promising novel molecules specific to a protein target. The source code of our implementation can be found at The DEL+JTVAE Package.

In future work, we will seek opportunities to validate our discoveries in wet-lab. To improve our framework, we will investigate more efficient and effective molecular fragmentation methods and incorporate these methods in our current and new AI for drug design approaches. We plan to test our approaches on more and larger datasets in drug design. Furthermore, we are interested in applying our approaches to other multi-objective design tasks.

## Data Availability

Publicly available datasets were analyzed in this study. This data can be found here: (ZINC database) https://zinc.docking.org/ (DrugBank) https://go.drugbank.com/.
